# Hair cell apoptosis and deafness in *Tmc1* mutations

**DOI:** 10.1073/pnas.2425215122

**Published:** 2025-03-18

**Authors:** Maryline Beurg, Dakota Elle Konrad, Robert Fettiplace

**Affiliations:** ^a^Department of Neuroscience, University of Wisconsin School of Medicine and Public Health, Madison, WI 53706

**Keywords:** mitochondria, hair cell, deafness, TMC1, apoptosis

## Abstract

Transmembrane channel-like protein 1 (TMC1), a pore-forming component of the mechano-electrical transducer (MET) channel in cochlear outer hair cells, is subject to numerous mutations causing deafness and hair cell death. We studied mice harboring semidominant mutations *Tmc1* p.T416K, p.M412K, and p.D569N, which all display functional MET channels at postnatal day (P)6 but become deaf by P21. Early signs of concomitant hair cell apoptosis were assayed in neonatal *Tmc1* mutants by labeling with Calcein-acetomethyl ester (AM), MitoTracker, and Annexin V, the latter labeling scramblase externalization of phosphatidyl serine. Reduced labeling with Calcein-AM was correlated with reduced MitoTracker, the targeting of mitochondria being confirmed with the uncoupling agent carbonylcyanide*p*-trifluoromethoxyphenylhydrazone, and use of MitoLight to monitor mitochondrial membrane potential. These markers demonstrated mitochondrial dysfunction in *Tmc1* mutants, even at P6 when MET currents were still present. Acoustic brainstem responses established that *Tmc1* p.D569N and *Tmc1* p.M412K mice were deaf by P15 and *Tmc1* p.T416K by P21. Two methods of blocking the stereociliary PMCA2 Ca^2+^ pump both elicited scramblase activity, suggesting that apoptosis is promoted by elevation of hair bundle [Ca^2+^]. Reduced PMCA2 density was found in the stereocilia of *Tmc1* mutants and was correlated with a decrease in MET channel Ca^2+^ permeability. Cre-Lox excision of the mutant M412K exon at P1 fully preserved hearing across all frequencies by P19 and promoted recovery to wild type of PMCA2 density. These results demonstrate that hair cells in *Tmc1* mutants have embarked on apoptosis at P6 and argue for connections between stereociliary PMCA2 density, hair cell apoptosis, and deafness.

## Significance

Mice with point mutations of Transmembrane channel-like protein 1 (TMC1), the putative mechanotransducer channel in auditory hair cells, become deaf by postnatal day P21. Nevertheless, these hair cell mutants exhibit functional transduction at P6, so the origin of deafness is obscure. However, even at this developmental stage, cochlear hair cells show signs of apoptosis, revealed by dysfunctional mitochondria and scramblase activity in the plasma membrane. We showed that scramblase is driven by blocking the plasma membrane calcium ATPase 2 (PMCA2) Ca^2+^ pump in the hair bundle, causing increased stereociliary Ca^2+^. Moreover, PMCA2 density is reduced in the mutants, which we suggest is the source of hair cell apoptosis and deafness. Decreased Ca^2+^ influx in TMC1 mutations may generate reduced PMCA2 density by a homeostatic mechanism.

Sensorineural hearing loss is a neurodegenerative disorder, where initial loss of cochlear hair cells causes deafness, a major functional disorder. A critical problem is the identity of the biochemical steps leading to cell death. Normal hearing occurs in the cochlea where sound-evoked mechanical vibrations are translated into electrical signals by the gating of mechano-electrical transducer (MET) channels in the hair cells (1). The pore-forming component of the hair cell MET channel is thought to be the transmembranechannel-like protein 1 (TMC1) protein (2–4), and there are at least 50 reported point mutations (pathogenic variants) of the *TMC1* gene causing human deafness (5), underscoring the key role of TMC1 in sound transduction. Previously described semidominant mouse (and human) mutations, *Tmc1* p.M412K, *Tmc1* p.T416K, and *Tmc1* p.D569N, are all linked to early hearing loss and hair cell degeneration (6, 7). While the phenotypic changes in MET channel properties in these *Tmc1* mutants have been well described (6, 8–11), the pathway leading to hair cell degeneration and death is unknown. And to confound the issue, large MET currents can still be observed in these mutants at P6 (12). Although mutant channels exhibit a reduced permeability to Ca^2+^, it is unclear whether this attribute contributes to hair cell degeneration (7).

The lipid bilayer of the plasma membrane contains multiple lipid types, including phosphatidyl choline, cholesterol, and phosphatidyl serine (PS), which are asymmetrically distributed between the internal and external monolayers. The acidic lipid PS is normally confined to the intracellular monolayer, where it is maintained by a flippase enzyme (13). However, under certain pathological conditions, lipid asymmetry is lost by activation of a scramblase, which causes PS externalization accompanying apoptosis and cell engorgement by macrophages (14, 15). The appearance of external PS can be visualized by treatment with Annexin V, membrane labeling with which has been reported in response to exposure to ototoxic aminoglycoside antibiotics like neomycin and streptomycin (16) and in certain *Tmc1* mutations (17). Those mutations are localized near the ion-conducting pore cavity according to modeling of the TMC1 structure (4, 18) and cryogenic-electron microscopy (cryoEM) structure of the TMC-1 complex in *Caenorhabditis elegans* (19). The modeling was based on the homology between TMC1 and TMEM16, a calcium-activated chloride channel. Importantly, TMEM16F, a homologous variant, is one of the main scramblase enzymes for the plasma membrane in many cell types, a feature that TMC1 may share (4, 18).

We have investigated the relationship between scramblase activity in *Tmc1* mutations and other early manifestations of apoptosis, especially mitochondrial dysfunction. The main route for intrinsic apoptosis involves the B-cell lymphoma 2 (BCL-2) path, recruiting proapoptotic factors BAK and BAX to permeabilize the mitochondrial membrane, leading to loss of mitochondrial membrane potential (20); there is subsequent release of cytochrome C into the cytoplasm and activation of caspase enzymes (21, 22). Accompanying these processes, damaged mitochondria are degraded by mitophagy, activated via multiple mechanisms. The aim of the work is to define the biochemical steps between *Tmc1* mutation, mitophagy, and hair cell death.

## Materials and Methods

### Mouse Mutants.

*Tmc1* p.D569N mutant mice were made by Applied StemCell Inc (Milpitas, CA) (23), and *Tmc1* p.T416K mice were made by Horizon Sage Labs Inc. (Saint Louis, MO), using CRISPR/Cas9 technology. For both genotypes, mutations were verified by 500 base-pair sequencing around the mutation site, and mice were subsequently bred for five generations, after which any off-target effects should have been eliminated. *Tmc1* p.M412K (*Beethoven*) mice were a gift of Walter Marcotti (Sheffield University, UK) originating from Karen Steel (Kings College London, UK). For all experiments apart from those involving Cre-Lox excision of M412K exon, measurements were performed mostly on homozygote C57Bl mice on a *Tmc2−/−* background to avoid complications arising from different channel properties of TMC2 compared to TMC1 (3, 24). *Tmc2−/−* knockout mice (B6.129S5-*Tmc2^tm1Lex^*/Mmucd) were obtained from the Mutant Mouse Regional Resource Center (University of California, Davis, CA). The original *Tmc1* p.M412K (*Beethoven*) mice were on a C3HeB/FeJ background but have been crossed multiple times with *Tmc2^−/−^* and are now effectively on C57Bl background. All three mutations used, identified by their common names, *Tmc1* p.M412K, p.D569N, and p.T416K lead to semidominant phenotypes (6, 7, 23).

Neonatal mice were killed by decapitation according to the animal protocol approved by the Institutional Animal Care and Use Committee at the University of Wisconsin-Madison. For all strains, a mixture of male and female mice was used and no gender-specific effects were noted. Acoustic brainstem responses (ABRs) were measured on P12 to P28 mutants and on wild-type mice, anesthetized with 10 mg/mL ketamine plus 1 mg/mL xylazine, and characterized using the Tucker-Davis Technology (Alachua, FL) Auditory Workstation. The effects of knocking out *Bac1* and *Bax* (Jackson labs, 006329; B6;129-*Bax^tm2Sjk^ Bak1^tm1Thsn^*/J), BCL-2 proteins that permeabilize the mitochondrial membrane during apoptosis, were investigated. Since *Bax^−/−^* is embryonically lethal, the *Bax^fl/fl^* was crossed with Pax2-Cre (25) to give expression in the cochlea (the mice were *Tmc1* p.D569N/+; *Bak−/−; Bax*^fl/fl^).

### Cre-lox Excision of the Mutant Exon.

The *Tmc1* p.M412K mutation was used because it is semidominant, so only one allele is needed to generate the deafness phenotype. Excision of the mutated *Tmc1* exon 13 containing M412K was achieved using *Tmc1 p.*M412K with Lox-P sites around exons 13 and 14. The knock-in mice were made by CRISPR/Cas9 by Applied StemCell, Milpitas, CA on a C57Bl background. They were crossed with ubiquitinC (UBC)-Cre-ERT2 mice (Jackson Labs 007001) to create *Tmc1* M412K/+; UBC-Cre-ERT2/+. The Cre was activated by injection of tamoxifen, 0.2 mg per g body weight intraperitoneally, initially at P5 and P6. To examine the effects of Cre-mediated recombination at an earlier age, tamoxifen was injected intraperitoneally at 0.2 mg per g body weight into the mother when the pups were P1 and P2. Following tamoxifen injection, the delay to Cre-mediated recombination took at least 7 d. The delay was determined by crossing UBC-Cre-ERT2 mice with Cre reporter Ai14 mice (Jackson Labs stock 007914: B6.Cg-Gt(ROSA)26Sor^tm14(CAG-tdTomato)Hze^/J), which exhibit tdTomato fluorescence following Cre-mediated recombination.

To verify the excision of exons 13 to 14 after Cre recombination of *Tmc1* M412K/+; UBC-Cre-ERT2/+ with tamoxifen, real-time PCR was performed by Transnetyx (Cordova, TN), using specific proprietary probes. Excision of exons 13 and 14 was confirmed, as well as the lack of the point mutation (M412K) at P19 on animals for which ABRs were recorded. In some instances, the exons were annotated as partial and incomplete, and those animals were noted in the present study. All breeder parents were also genotyped to verify their homozygosity for *Tmc1* M412K ^fl/fl^ before being crossed with UBC-Cre-ERT2 mice. The offspring neonates were genotyped prior to injection of tamoxifen to verify that no exon deletion had occurred (i.e., the Cre was not leaky), and all pups were confirmed to be *Tmc1* p.M412K ^fl/+^; Cre hemizygous. The sequence of the probes used for UBC-Cre-ERT2 is available on the JAX protocol (https://www.jax.org/strain/007001). Since *Tmc1^+/−^* heterozygotes yield the same MET current and phenotype as *Tmc1^+/+^* homozygotes (3), *Tmc1* does not show haploinsufficiency; therefore, in the absence of the mutant M412K allele, the wild-type allele gives the wild-type phenotype and should rescue the deafness phenotype. It was not possible to immunolabel for the protein lacking exons 13 and 14, distinguishing it from the full-length protein, so we cannot determine whether the truncated protein after excision by Cre recombination was expressed. However, the exons removed code for a major portion of the proposed channel pore (7, 18), so even were the truncated protein to be expressed, it would be unlikely to affect transduction. Hearing levels were determined between P12 and P28 using ABRs.

### Determination of Apoptosis.

The early apoptotic state of the cochlear outerhair cells (OHCs) was determined in isolated apical turn organs of Corti of P4 to P7 homozygote mutant mice, bathed in Hanks’ Balanced Salt Solution (HBSS) in the presence of various indicators at room temperature (21 to 23 °C). Indicators were usually perfused over the preparation for 30 min, which was then washed in HBSS and fixed in 4% paraformaldehyde plus 2.5 mM CaCl_2_ for 10 min at room temperature, washed, and processed as previously described (23). Preparations were mounted on coverslips with Fluoromount-G (Southern Biotech, Birmingham, AL) and viewed with a 60× oil-immersion Planapochromat (NA = 1.4) on a Nikon A1 laser-scanning confocal microscope; 0.5 to 1.0 µm sections were acquired along the cell length, and fluorescence intensity was quantified with ImageJ (Fiji).

Early signs of hair cell apoptosis were detected using three indicators: i) Calcein-AM (Biotium, Fremont, CA) dissolved in HBSS at 10 µM according to the manufacturer’s protocol. The colorless acetoxymethyl AM ester diffuses across the plasma membrane and is then de-esterified in the cytoplasm to yield a green fluorescence in healthy cells but not in proapoptotic cells (26). Perfusion with Calcein-AM required 30 min to achieve a good cytoplasmic signal. ii) Annexin V coupled to Alexa Fluor 568 or Alexa Fluor 488 (Invitrogen, ThermoFisher, Waltham, MA) at a 1:50 dilution in the bathing solution was used to assay for PS externalization (16); in healthy cells, PS is exclusively located on the inner membrane, but its distribution equalizes between inner and outer leaflet in apoptotic cells (14). iii) MitoTracker-Orange CM-H_2_ TMRos M7511 (ThermoFisher, 1.0 µM) detected the presence of active mitochondria, and the fluorescent signal was taken to indicate mitochondrial density and health. In some experiments, the MET channel blocker dihydrostreptomycin (DHS; Sigma-Aldrich, St. Louis, MO), or the mitochondrial uncoupling agent carbonylcyanide *p*-trifluoromethoxyphenylhydrazone (carbonyl cyanide-p-trifluoromethoxyphenylhydrazone (FCCP); Sigma-Aldrich, 2 µM) were also added to the bathing solution. In view of the variation in half-blocking concentrations of DHS for the different mutants (7, 10), two DHS concentrations were applied, 0.1 and 1.0 mM, the latter exceeding all the DHS half-blocking concentrations for the mutants. Mitochondrial membrane depolarization was monitored with MitoLight (Chemicon APT 142; Sigma-Aldrich), which was dissolved in the kit buffer and diluted 1:100 in HBSS according to the manufacturer’s protocol. MitoLight, unlike Mito tracker, does not survive fixation and permeabilization of the cell so the red mitochondrial fluorescence was followed over 30 min with live imaging on a Leica upright confocal microscope viewed with a Leica 63× 0.9 NA water immersion objective. For these experiments, cochlear preparations were isolated in saline containing 1 mM Trolox as an antioxidant. Cells were also treated with both MitoTracker and Annexin V, and labeling was followed over 30 min to monitor the effects of blocking the plasma membrane calcium ATPase (PMCA2) Ca^2+^ pump. PMCA2 block was achieved (27) either by raising the extracellular pH to 9.0 in saline buffered with 10 mM N-tris(hydroxymethyl)methyl-4-aminobutanesulfonic acid (TABS) (Sigma-Aldrich), or with the membrane-permeant inhibitor carboxyeosin diacetate succinimidyl ester (Invitrogen Life Sciences, Carlsbad, CA; 50 µM). These agents were added to normal saline and cells were counterstained with MitoTracker. Annexin V labeling of PS requires extracellular Ca^2+^ with a binding constant, K_D_, of between 50 and 100 µM (28, 29). Therefore, to test the effects on Annexin V labeling of Ca^2+^ influx during PMCA2 inhibition, extracellular Ca^2+^ was reduced from 1.5 to 0.5 mM, a concentration well above the K_D_ that will diminish Ca^2+^ influx.

### Immunolabeling.

Immunofluorescence labeling for PMCA2 was performed as described previously (30), using an affinity-purified rabbit polyclonal antibody (NR2; ThermoFisher Scientific, Pittsburgh, PA). P5 to P7 mice were decapitated, and isolated cochleas were fixed in 4% paraformaldehyde in phosphate buffer for 30 min at room temperature, then treated with 0.5% TritonX-100 and blocked with 10 percent goat serum for 1 h. Isolated cochlear coils were incubated overnight at 4 °C with the primary NR2 antibody [ThermoFisher Scientific, see refs. 30 and 31 at 1:400 dilution], and subsequently labeled with Alexa Fluor 488 goat anti–rabbit IgG secondary antibody followed by Alexa Fluor 568 phalloidin (Invitrogen) to label bundle actin. Mounted tissue was viewed under a 60× (NA = 1.4; ELWD Planfluor; Nikon) oil-immersion objective in a Nikon A1 laser scanning confocal microscope. For the PMCA2 measurements, the confocal depth was 0.6 µm, less than the hair bundle height, about 3 to 4 µm in apical OHCs of both the wild type and mutants. Fluorescence intensities were measured using ImageJ software for 50 or more OHC bundles chosen at random in each preparation. For PMCA2 immunolabeling of older animals (30), P16 and P19 mice were anesthetized with isoflurane, decapitated, and the cochlea rapidly isolated. A small hole was made in the cochlear apex and the tissue fixed in 4% paraformaldehyde in phosphate buffer for 1 h at room temperature, then washed and decalcified in 120 mM Na ethylene diamine tetra-acetic acid, pH 7.4, for 12 h. Cochlear coils were dissected out, processed, and immunolabeled as for young mice (see above), then mounted and viewed in a Nikon A1 laser scanning confocal microscope. Butylhydroquinone, a blocker of the sarcoplasmic and endoplasmic calcium (SERCA) ATPase, was obtained from Enzo, Farmingdale, NY).

### Statistical Test.

All results are quoted, unless otherwise indicated, as means ± 1 SD and statistical tests used a two-tailed *t* test; significance tests indicated in figures as *P < 0.05, **P < 0.01, and ***P < 0.001. ABRs and immunological labeling were performed on at least three mice for each mutation.

## Results

### Annexin V Labeling in *Tmc1* Mutants.

**Fig. 1.**
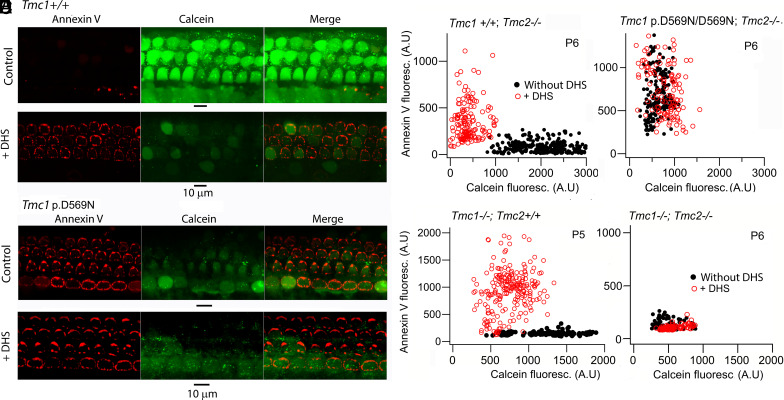
Effects of DHS on Annexin V and Calcein-AM labeling. (*A*) Surface view of the cochlear apex of *Tmc1+/+* mouse showing control label for Calcein-AM (green). Treatment with DHS (0.1 mM, 30 min) elicits Annexin V label (red) and loss of Calcein-AM. (*B*) In *Tmc1* p.D569N homozygote, Annexin V label but little Calcein-AM is present without DHS. (*C*) OHC fluorescent intensity counts from *Tmc1^+/+^; Tmc2^−/−^* show nonoverlap with and without DHS. (*D*) In *Tmc1* p.D569N/D569N homozygote mice, OHC fluorescent intensity counts overlap with and without DHS indicating apoptosis is already present in untreated mice. (*E*) OHC counts from *Tmc1^−/−^; Tmc2^+/+^* show nonoverlap with and without DHS, like *Tmc1^+/+^; Tmc2^−/−^,* indicating either TMC1 or TMC2 can support scramblase activity. (*F*) No Annexin V label seen in *Tmc1^−/−^; Tmc2^−/−^,* though the resting Calcein-AM counts are much smaller than controls. Each spot is one OHC and the plots combine results from three cochleas. Ordinate scale “AU” indicates arbitrary units, with measurements in all conditions being performed under the same confocal settings.

We examined scramblase activity in three homozygous *Tmc1* mutants, *Tmc1* p.D569N; D569N, *Tmc1* p.M412K: M412K, and *Tmc1* T416K; T416K. Despite the mutation, the MET current developed over the first few postnatal days, reaching a maximum amplitude around P6 (7, 23). Nevertheless, mice of all three mutations were deaf by P28 (7), raising the question about whether there was any earlier dysfunction. We therefore searched for early signs of hair cell apoptosis using Annexin V, a scramblase marker, and Calcein-AM, an indicator of cytoplasmic dysfunction. We focused on OHCs at P6, first showing that Annexin V labeling occurred in *Tmc1+/+* homozygotes on perfusion with the MET channel blocker, DHS, as originally demonstrated (16, 17) (Fig. 1 *A* and *B*). The development of the Annexin V label, prominent on the lateral membrane around the cuticular plate and associated with membrane blebbing (32), was accompanied by a decrease in Calcein-AM label. A 30 min. exposure was needed to obtain a clear and reproducible Calcein-AM signal by which time the Annexin V had spread from the hair bundle to the periphery of the apical cell membrane. Following DHS perfusion, the Annexin V label in *Tmc1+/+* increased from 84 ± 8 arbitrary units (AU) counts to 345 ± 16 counts and the Calcein-AM label decreased from 2,041 ± 36 AU counts to 405 ± 18 AU counts. In the *Tmc1* p.D569N mutant, the Annexin V and Calcein-AM points with and without DHS treatment were overlapping. Two DHS concentrations were applied, 0.1 and 1.0 mM, and gave similar results. Thus, Annexin V labeling in *Tmc1* p.D569N control (without DHS) gave 622 ± 13 counts (N = 191); in *Tmc1* p.D569N plus 0.1 mM DHS, counts were 729 ± 26 (N = 122), whereas in *Tmc1* p.D569N plus 1.0 mM DHS, counts were not significantly different at 669 ± 22 (N = 175) (*t* test, *P* = 0.55). Hereafter, all counts are specified as AU, with imaging done in each set of experiments under the same confocal settings.

The Annexin V label was present after DHS treatment in the single knockouts *Tmc1+/+; Tmc2−/−* and in *Tmc1−/−; Tmc2+/+* (Fig. 1 *C* and *E*), but not in the double knockout, *Tmc1−/−;Tmc2−/−* (Fig. 1*F*). This suggests that scramblase activity requires either one of TMC1 or TMC2, a conclusion disagreeing with ref. 17, who found TMC2 would not support scramblase activity. In the double knockout, *Tmc1−/−; Tmc2−/−,* a reduction in Calcein-AM labeling was seen (454 ± 13 AU, Fig. 1*F*), significantly smaller than that for *Tmc1+/+; Tmc2−/−* (2,041 ± 36 AU; Fig. 1*C*) or for *Tmc1−/−; Tmc2+/+* (1,217 ± 31 AU; Fig. 1*E*) implying that PS externalization is not obligatory for the hair cell to embark on the apoptotic pathway.

**Fig. 2.**
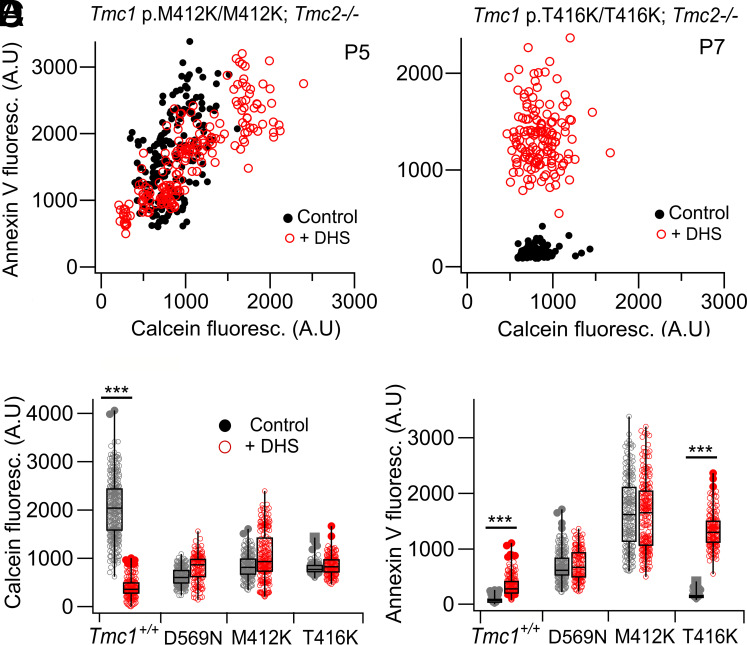
Effects of DHS on Annexin V and Calcein-AM labeling in two other *Tmc1* mutants. (*A*) In *Tmc1* p.M412K homozygote mice, OHC counts with and without DHS overlap indicating apoptosis already present in mice before DHS treatment. (*B*) OHC counts from *Tmc1* p.T416K show nonoverlap of Annexin V label with and without DHS even though the Calcein-AM counts are reduced. (*C*) Bar plots for Calcein-AM labeling for *Tmc1 +/+, Tmc1* p.D569N/D569N homozygotes, *Tmc1* p.M412K/M412K homozygotes and *Tmc1* p.T416K/T416K homozygotes. (*D*) Bar plots for Annexin V labeling showing differences after DHS treatment for *Tmc1^+/+^* and *Tmc1* p.T416K, but not *Tmc1* p.M412K.

Labeling with Annexin V and Calcein-AM was seen in the other two mutants (Fig. 2). In homozygous *Tmc1* p.M412K/M412K, label after DHS treatment overlaid that in the absence of the antibiotic, but for the *Tmc1* p.T416K/T416K, the labeling clouds with and without DHS were distinct (Fig. 2 *A* and *B*). In the latter mutant, there was an increase in Annexin V label with DHS (Fig. 2*D*), suggesting that the mutant phenotype at this stage is less severe than that observed in both *Tmc1* p.M412K and *Tmc1* p.D569N. Based on levels of Annexin fluorescence in the *Tmc1* mutant relative to the *Tmc1^+/+^* wild type in the absence of DHS, the mutants are ordered M412K > D569N > T416K. Annexin V labeling in the *Tmc1* p.D569N mutant without DHS is smaller than for *Tmc1* p.M412K and may be underestimated due to a 40 percent reduction in TMC1 expression in *Tmc1* p.D569N, which decreases the number of MET channels (33), and hence scramblase activity. If the 40 percent reduction in MET channel numbers is factored into the calculation of Annexin fluorescence in the *Tmc1* D569N mutant relative to the *Tmc1^+/+^* wild type, then the order of mutant severity becomes D569N > M412K > T416. For all three mutants, there is a decrease in MET channel Ca^2+^ permeability, the reduction relative to the Tmc1+/+ wild type being D569N (0.29), M412K (0.35), and T416K (0.66) (12).

### Mitochondrial Dysfunction.

**Fig. 3.**
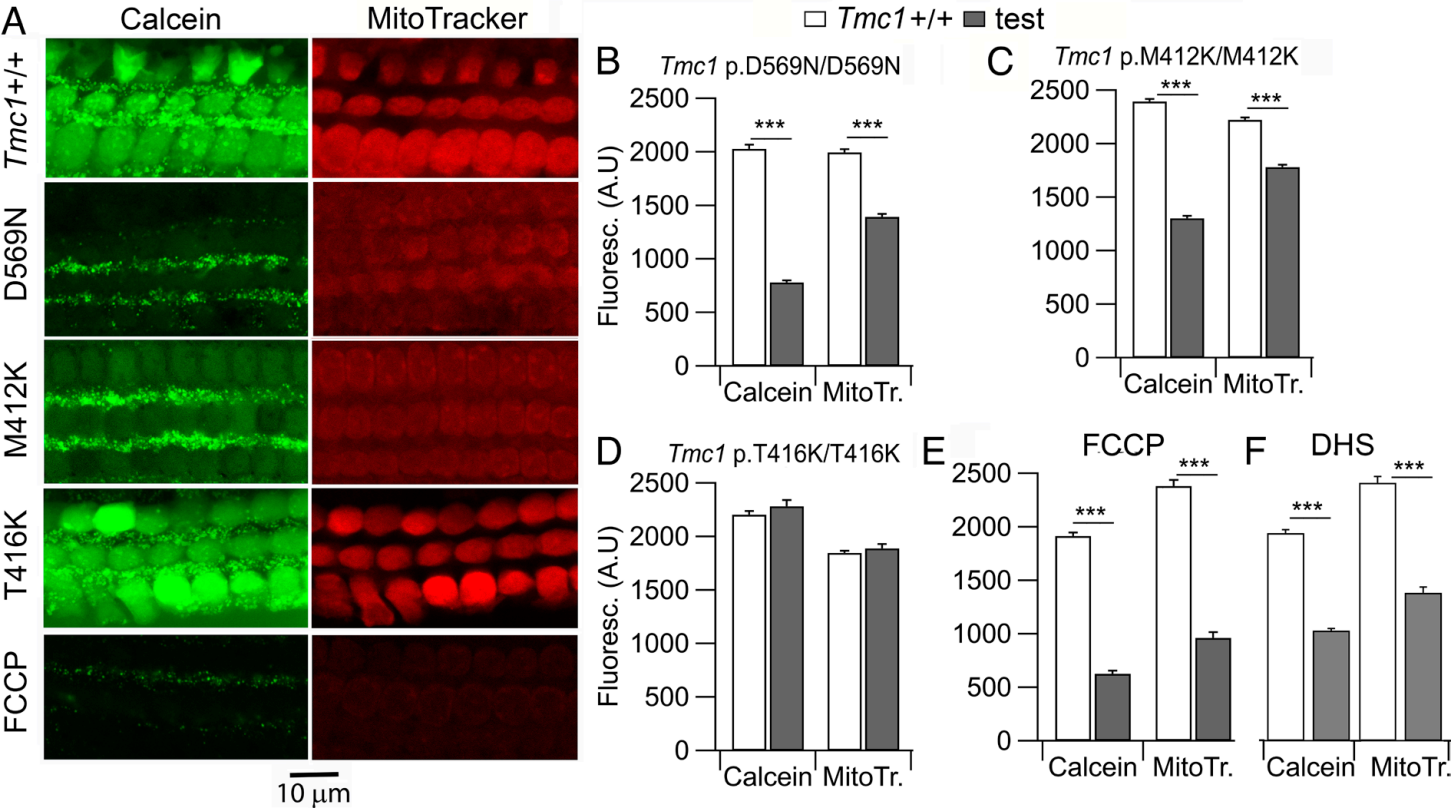
Comparison of Calcein-AM and MitoTracker label in *Tmc1* mutants. (*A*) Surface views of the organ of Corti, showing three OHC rows with reduction in Calcein-AM and MitoTracker labeling in *Tmc1* p.D569N/D569N and *Tmc1* p.M412K/ M412K homozygotes and *Tmc1*^+/+^ after FCCP. (*B*) Bar plots quantifying significant reduction in label for *Tmc1* p.D569N; D569N. (*C*) Bar plots showing comparable reduction in label for *Tmc1* p.M412K/M412K. (*D*) Bar plots showing no significant reduction in label for *Tmc1* p.T416K; T416K. (*E*) Bar plots in *Tmc1*^+/+^ mice showing significant reduced labeling after treatment with the mitochondrial uncoupling agent FCCP; changes in Calcein-AM and MitoTracker labels indicate apoptosis due to mitochondrial dysfunction. (*F*) DHS treatment (0.1 mM, 30 min) in *Tmc1*^+/+^ mice causes a significant decrease in labeling.

Mitochondria are distributed throughout the hair cell soma, often congregated beneath the cuticular plate underlying the hair bundle, and serve multiple functions, including energy production (ATP) and Ca^2+^ buffering (27). Mitochondrial function in the *Tmc1* mutants was studied using MitoTracker, a lipophilic cationic dye that accumulates in the mitochondria dependent on their negative membrane potential and then forms covalent bonds in the mitochondrial matrix (34, 35). MitoTracker was included in the bathing solution along with Calcein-AM, and there was good correlation in the overall level of cellular labeling with both markers in all three mutants (Fig. 3 *A*–*D*). A significant reduction in both labels at P6 was observed in *Tmc1* p.D569N and *Tmc1* p.M412K homozygotes compared to wild-type *Tmc1+/+*. However, no reduction in labeling for either marker was seen in *Tmc1* p.T416K. The mitochondrial target was confirmed by exposure to the uncoupling agent FCCP to cause loss of mitochondrial membrane potential, which produced similar results to those evident in *Tmc1* p.D569N (Fig. 3 *B* and *E*). A decrease in MitoTracker was also seen after DHS treatment that led to scramblase activity (Fig. 3*F*). It is thought that the consequences for hair cell apoptosis of DHS are partly attributable to the effects of the antibiotic on mitochondrial integrity and energy production (22).

**Fig. 4.**
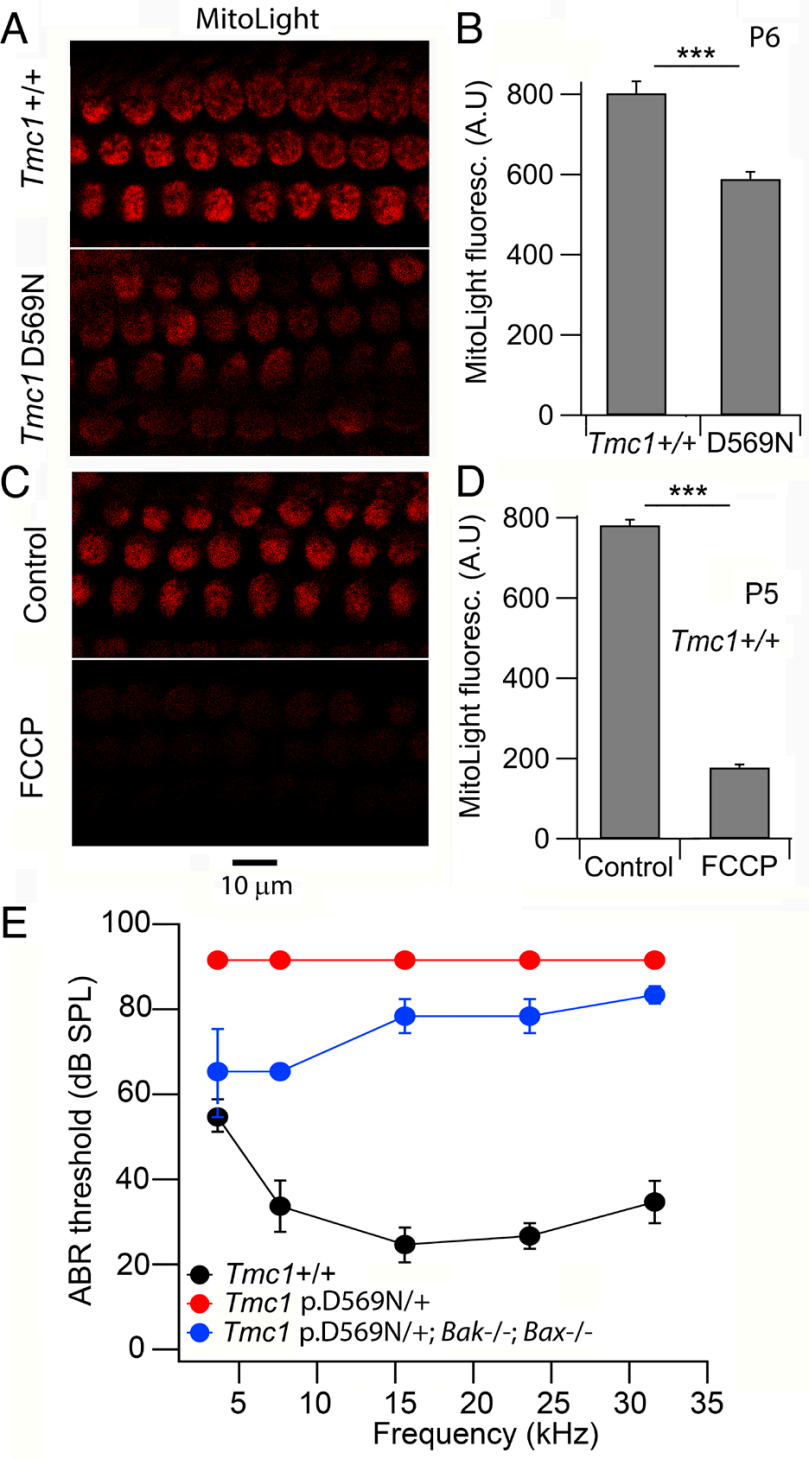
MitoLight live imaging of mitochondrial membrane potential. (*A*) Surface view of OHCs in the organ of Cortis of control *Tmc1^+/+^* and *Tmc1* p.D569N/D569N under the same imaging conditions. (*B*) Bar plots of reduction in MitoLight label in *Tmc1* mutant. (*C*) OHCs in the organ of Cortis of control *Tmc1*^+/+^ without and with application of 20 µM FCCP. (*D*) Bar plots of significant reduction in MitoLight label with FCCP. (*E*) Effects of *Bak; Bax* knockouts on ABR thresholds in a *Tmc1* mutant. ABRs at P28 in *Tmc1* p.D569N/+ heterozygotes (red circles; mean ± SD; N = 4) show a 70 dB threshold elevation compared to *Tmc1^+/+^* (black circles; mean ± SD; N = 5). Double knockout *Bak; Bax* (causing mitochondrial depolarization) on *Tmc1* D569N/+ (blue circles; mean ± SD; N = 2) produces up to 30 dB threshold improvement.

Further confirmation of the mitochondrial dysfunction mechanism was obtained by labeling with MitoLight, a cationic dye that assays mitochondrial membrane potential (36). Since the MitoLight label is lost on cellular permeabilization and fixation, labeling was followed using live imaging, which revealed a significant reduction in MitoLight label in *Tmc1* p.D569N/D569N homozygotes compared to *Tmc1+/+* (Fig. 4 *A* and *B*). A reduction in MitoLight label was also seen after treating *Tmc1+/+* with the uncoupling agent FCCP (Fig. 4*C*), an ionophore which also decreases mitochondrial membrane potential. From these results, we conclude that during the triggering of apoptosis in the *Tmc1* mutants, one major pathway occurs through mitochondrial dysfunction. The decrease in MitoTracker label may indicate a reduction in the mitochondrial density, normally referred to as mitophagy. Furthermore, at P6, the reduction in both labels was graded according to the mutant, with *Tmc1* p.D569N being the most affected and *Tmc1* p.T416K the least (Fig. 2 *B*–*D*). Thus, for MitoTracker, the ratio of mutant over wild-type *Tmc1^+/+^* control was 0.7 ± 0.20 (D569N), 0.8 ± 0.24 (M412K) and 1.0 ± 0.23 (T416K); for Calcein-AM, the ratios were 0.38 ± 0.19 (D569N), 0.54 ± 0.20 (M412K) and 1.0 ± 0.20 (T416K). This gradation echoes the variation in Ca^2+^ permeability reported. We assume that an effect in *Tmc1* T412K would have been seen if labeling had been followed to later postnatal times. In support of the apoptotic role of mitochondria, we found that conditional knockout of *Bak* and *Bax* caused a 20 to 30 dB improvement in acoustic threshold, especially in the low-frequency register (Fig. 4*E*). BAK and BAX are proteins recruited during apoptosis to permeabilize the mitochondrial membrane, thereby depolarizing the mitochondrial membrane potential (20, 37).

### ABR Monitoring of Hearing Function.

**Fig. 5.**
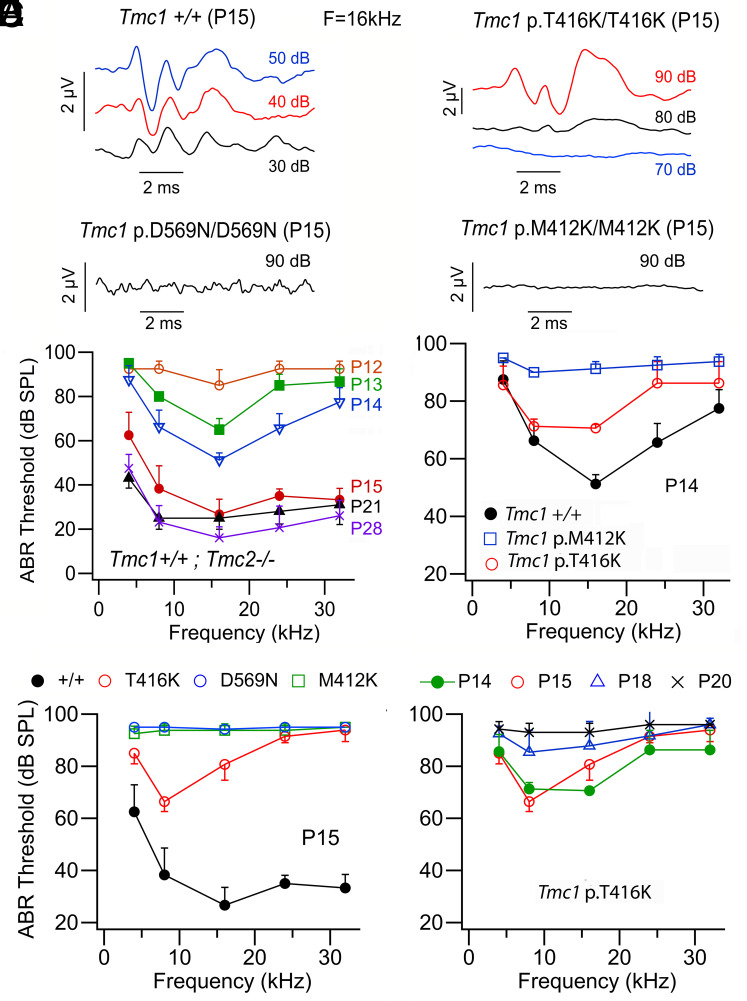
ABR in mice after the onset of hearing. (*A*) ABR records at P15 in *Tmc1*^+/+^ (*Top*) and in *Tmc1* p.D569N/D569N (*Bottom*) which gives no response at 90 dB SPL; (*B*) ABR records at P15 in *Tmc1* p.T416K; T416K (*Top*) and in *Tmc1* p.M412K/M412K (*Bottom*) which gives no response at 90 dB SPL. (*C*) ABR thresholds for *Tmc1*^+/+^ showing the development of hearing sensitivity between P12 and P28. (*D*) ABR thresholds at P14 for *Tmc1* p.M412K/M412K and for *Tmc1* p.T416K/T416K homozygotes which retain some hearing. (*E*) ABR thresholds at P15 for *Tmc1* p.M412K; M412K, *Tmc1* p.D569N; D569N (both deaf) and *Tmc1* p.T416K; T416K which has low-frequency hearing. (*F*) ABR thresholds for *Tmc1* p.T416K/T416K showing loss of hearing between P14 and P20. For each plot (*C*–*F*), each point represents the mean value for four mice.

ABRs were used to chart the loss of hearing in the three mutants (Fig. 5 *A*–*F*). The onset of hearing in mice occurs around P12 (38, 39). It is possible to measure ABRs from P12 onward and over the subsequent week, the threshold improves, and the high-frequency hearing range expands to achieve adult performance by P28 (Fig. 5*C*). Neither mutant, homozygous *Tmc1* p.D569N nor *Tmc1* p.M412K displays hearing at P15 (Fig. 5 *D* and *E*). In contrast, some low-frequency hearing persists in homozygous *Tmc1* p.T416K mutant at P14 and P15, and hearing across the frequency range does not disappear in this mutant until P20 (Fig. 5*F*). This result is consistent with direct measures of transduction in the *Tmc1* p.T416K mutant assessed by permeability of the MET channel to the fluorescent dye FM1-43, transduction being lost in inner hair cells by P20 (7). The onset of deafness in the three mutants correlates well with the indications of hair-cell malfunction using the early markers of apoptosis.

**Fig. 6.**
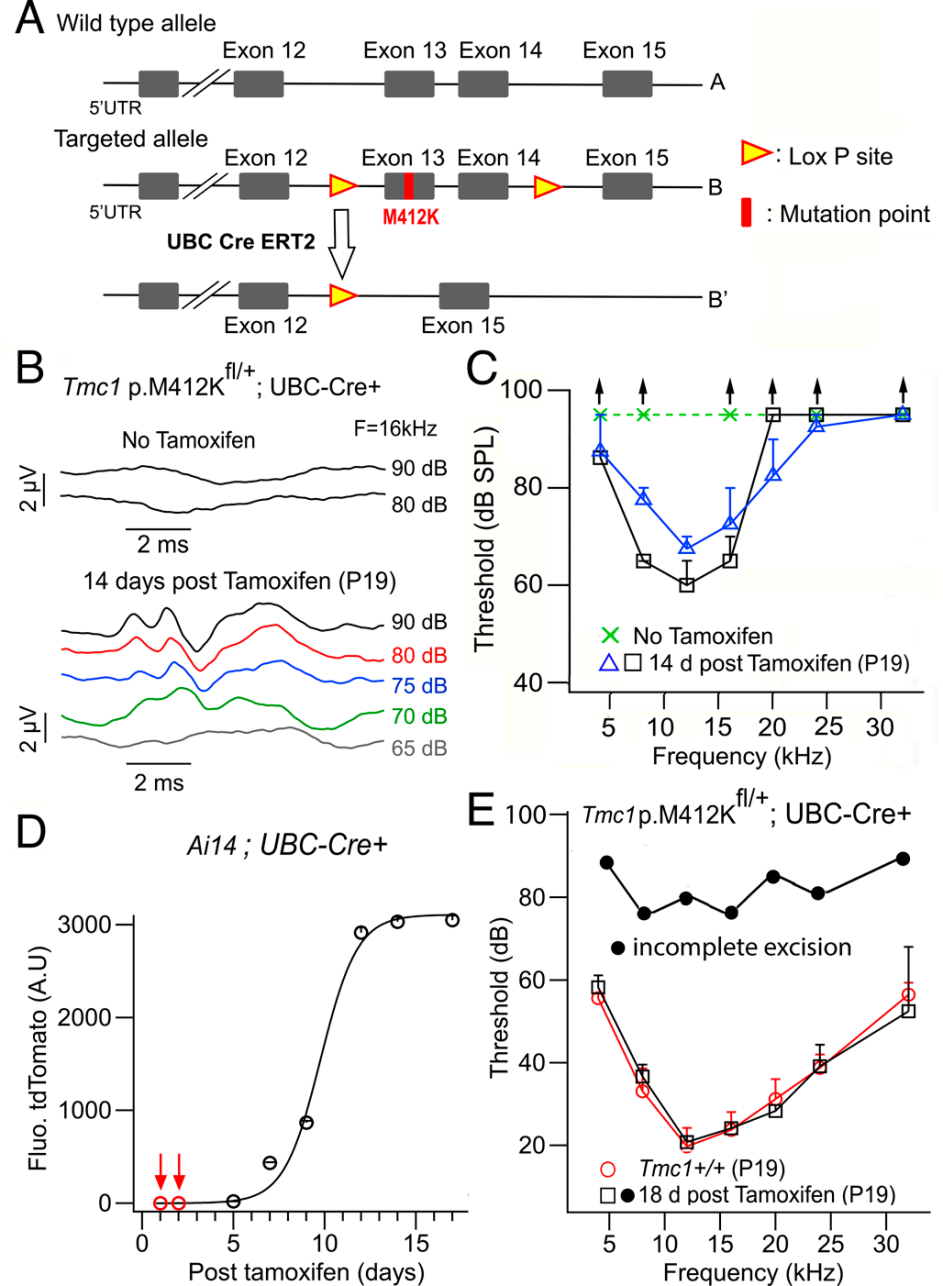
Excision of *Tmc1* mutant exon 13 containing M412K mutation to reverse hearing loss. (*A*) Schema of technique involving Lox-P sites around exons 13 and 14; activation of UBC-Cre-ERT2 after tamoxifen injection removes exon 13 (and 14) causing the mutant allele not to be expressed, leaving only the wild-type allele. (*B*) ABR thresholds of P19 *Tmc1* p.M412Kfl/+; UBC-Cre/+ mouse without tamoxifen (above), and P19 *Tmc1* p.M412Kfl/+; UBC-Cre/+ mouse following tamoxifen injection at P5 (below). (*C*) ABR thresholds showing some low-frequency hearing: *Tmc1* M412K at P19 (squares and triangles) in two mice with tamoxifen injection at P5 and P6; *Tmc1* M412K without tamoxifen (green crosses). The threshold for the wild-type control *Tmc1^+/+^* is shown in *E*. (*D*) Time course of Cre -activation determined from tdTomato fluorescence of the Cre reporter, with Tamoxifen injected at P1 and P2; half-activation occurred 10 d postinjection. (*E*) ABR threshold showing full preservation of hearing across the frequency range for *Tmc1* M412K (P19, squares; mean ± SEM, N = 2) and without hearing (P19, filled circles, incomplete excision, N = 1) with tamoxifen injection at P1 and P2; control *Tmc1^+/+^* (P19, red open circles; mean ± SEM, N = 4).

Hearing loss is localized to the third postnatal week, so we wished to explore whether the degenerative mechanisms involved were reversible. To achieve this, we undertook conditional removal of the mutant exon for the semidominant mutation *Tmc1* p.M412K. A somewhat similar approach has been used to reverse the effects of the *Spns2* mutation (40), which causes loss of endolymphatic potential and deafness. Mice were created with targeted mutations of Lox-P sites around the exon 13 (and 14) containing the mutant sequence (Fig. 6*A*). Both exon 13, containing the M412 mutation, and exon 14 were enclosed by the LoxP sites because the intron sequence between the two exons was small. These mice were then bred with transgenics expressing a Cre-ERT2 fusion gene under the control of the human UBC promoter. Double heterozygotes, *Tmc1* p.M412K/+; UBC-Cre-ERT2/+, were selected and given two tamoxifen injections to activate the Cre and remove the mutant exon (Fig. 6*A*). Exon removal after tamoxifen was confirmed by RT PCR (*Materials and Methods*). In the first round, tamoxifen was injected at P5 and P6, an age when there were manifestations of hair cell dysfunction; mouse hearing was then assayed 14 d after the first injection, at P19. Three mice were studied, two of which gave partial improvement in hearing confined to low frequencies (Fig. 6 *B* and *C*), and the third was deaf. However, the best thresholds were 40 dB above the ‘no tamoxifen’ controls, and the frequency range affected was confined to the apical turn, with characteristic frequencies of 4 to 20 kHz (41). Since the Cre-mediated recombination took more than a week, it is possible that by P12 (7 d after the first tamoxifen injection), the high-frequency hair cells had deteriorated too far to be reversible. We determined the time to Cre recombination using a tdTomato Ai14 Cre-reporter (*Materials and Methods*) and found a half-time for Cre-activation of 10 d (Fig. 6*D*). We subsequently performed tamoxifen injections at P1 and P2 on other mice, three of which survived until ABR testing. The earlier injection caused better preservation of hearing thresholds over a broader frequency range in two of the mice but the third was deaf (Fig. 6*E*). The variation is probably attributable to incomplete exon 13 to 14 excision in the third mouse, as determined by real-time PCR.

### Hair Cell Dysfunction Mediated by an Increase in Intracellular Ca^2+^.

**Fig. 7.**
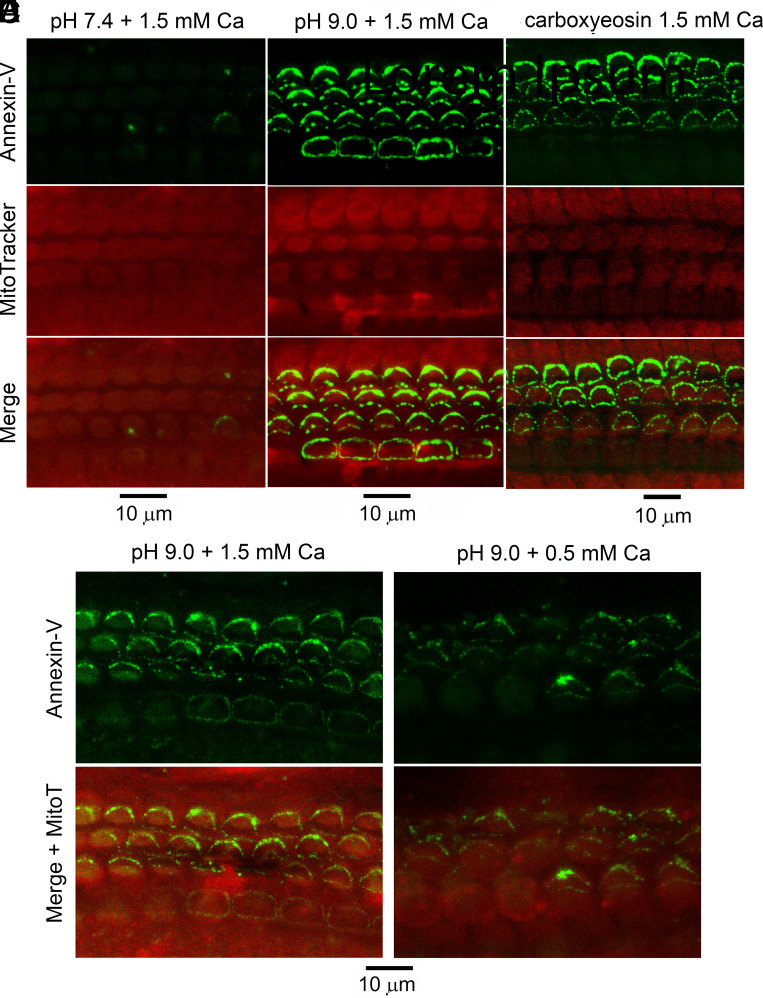
Inhibition of PMCA2 pumps elicits Annexin V labeling. (*A*) Apical cochlear whole mount at pH 7.4; (*B*) PMCA2 inhibition by raising extracellular pH to 9.0 preventing Ca^+^/H^+^ exchange, to cause Annexin V labeling (green) at normal (1.5 mM) extracellular Ca^2+^. (*C*) PMCA2 inhibition with carboxyeosin (50 µM, 30 min) causes Annexin V labeling (green) at normal (1.5 mM) extracellular Ca^2+^. (*D*) PMCA2 inhibition by raising extracellular pH to 9.0 in a separate experiment causes Annexin V labeling (green) at normal (1.5 mM) extracellular Ca^2+^. (*E*) PMCA2 inhibition by raising extracellular pH to 9.0 causes diminished Annexin V labeling (green) in 0.5 mM extracellular Ca^2+^. For both sets of experiments (*B* and *C*) and (*D* and *E*), similar results were obtained in one other organ of Corti. For all experiments, MitoTracker was used to visualize OHCs (red). Observations were made on apical organs of Corti from P6 *Tmc1^+/+^*; *Tmc2^−/−^* mice.

One of the main proteins required for PS exposure in the plasma membrane of apoptotic cells is TMEM16F, a scramblase regulated by elevated Ca^2+^ (14, 42, 43). TMEM16F is homologous to the (nonscramblase) TMEM16A, which itself is homologous to TMC1 (4, 18). There has been no definitive evidence on Ca^2+^ being able to activate the scramblase in hair cells although considerable effort was expended to test its involvement by altering hair cell intracellular Ca^2+^ (16). Cytoplasmic Ca^2+^ depends in the long term on the balance between influx of the ion through various ion channels, and its efflux, mediated by a PMCA2 calcium pump. One factor limiting changes in intracellular Ca^2+^ may be the PMCA2 which is present at high density in the OHC stereociliary membrane (30, 44, 45). We aimed to alter stereociliary Ca^2+^ by blocking PMCA2, a procedure we have previously shown to increase OHC Ca^2+^ (27). Two methods were employed in cochleas from homozygous Tmc1^+/+^ mice: block with carboxyeosin and raising the extracellular pH, which impedes the Ca^2+^/H^+^ exchange required for Ca^2+^ extrusion (27). Both methods were applied during live imaging in conjunction with Annexin V labeling. No immediate response was seen, but after 20 to 30 min, the Annexin V label appeared when performed in high (1.5 mM) extracellular Ca^2+^ (Fig. 7 *A* and *B*). However, when Ca^2+^ influx through the MET channels decreased in low (0.5 mM) extracellular Ca^2+^, Annexin V labeling was reduced 2.3-fold. Use of both blocking methods was performed three times, all with the same outcome. A small but significant increase (2.8 times over background; N = 85, *t* test *P* < 0.001).in Annexin V labeling was also seen on treatment with 0.1 mM butylhydroquinone, a blocker of the endoplasmic reticulum ATPase, which had previously been shown to increase hair cell cytoplasmic Ca^2+^ (46). For comparison, perfusion with saline at pH 9.0 increased the counts 11.9 times over the control, pH 7.4 (N = 162; Fig. 7 *A* and *B*). Treatment with carboxyeosin increased the counts 7.8 times over the same control, pH 7.4 (N = 110; Fig. 7 *A* and *C*). Together, these results argue that the PS externalization is driven by an increase in stereociliary Ca^2+^ (47).

**Fig. 8.**
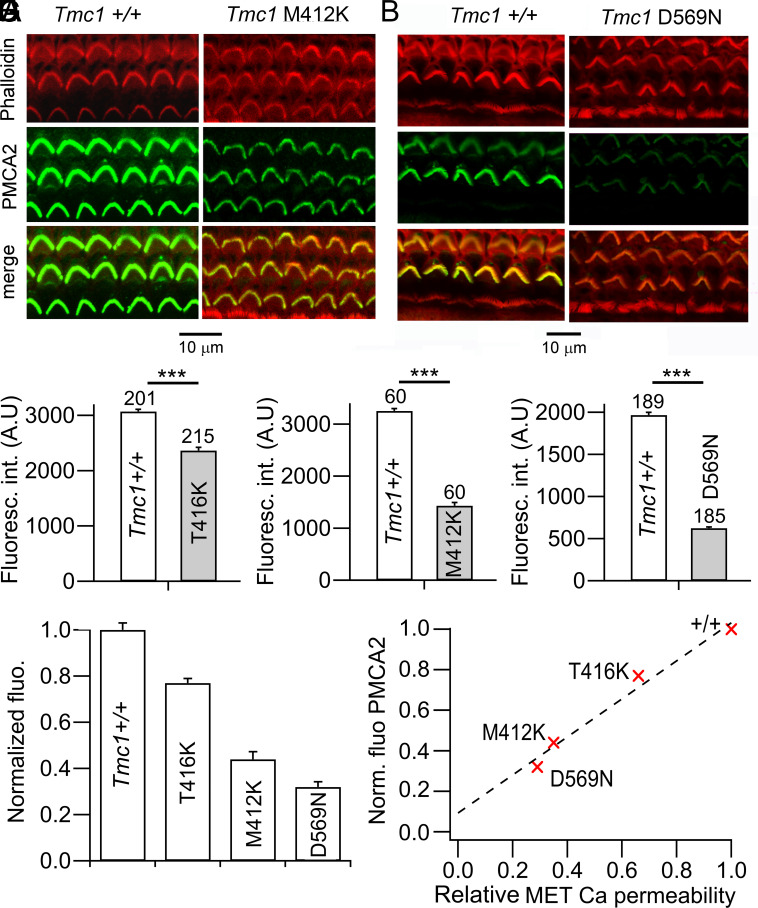
Reduced expression of PMCA2 pumps in *Tmc1* mutants. (*A*) Immunofluorescence of OHCs in apical turn labeled with anti-PMCA2 antibody for *Tmc1^+/+^* (*Left*) and homozygote *Tmc1* p.M412K/M412K (*Right*). Hair bundle actin labeled with phalloidin. (*B*) Apical OHCs labeled with anti-PMCA2 antibody for homozygous *Tmc1^+/+^* (*Left*) and *Tmc1* p.D569N/D569N (*Right*). (*C*) Collected measurements of OHC bundle fluorescence intensity in *Tmc1^+/+^* (closed bars) and *Tmc1* p.T416K (gray). (*D*) Collected measurements of OHC bundle intensity in *Tmc1+/+* (closed bars) and *Tmc1* p.M412K (gray). (*E*) Collected measurements of OHC bundle intensity in *Tmc1+/+* (closed bars) and *Tmc1 p.*D569N (gray). Numbers of bundles assayed given above each bar. (*F*) Comparison of PMCA2 reduction in the three mutants, obtained by normalizing controls in each mutant to 1.0. (*G*) Correlation between PMCA2 density from *F* and the decrease in Ca^2+^ permeability of the MET channel (12). The correlation coefficient for the linear fit is 0.94. Immunofluorescence measurements were performed on pairs of P6 mice.

Given the impact of PMCA2 inhibition on scramblase activity, we hypothesized that defects in the PMCA2 calcium pump might underlie the deleterious consequences of the *Tmc1* mutations. For example, it is known that point mutations in PMCA2 can evoke hair cell death and deafness in *Oblivion* (48) and in *Tommy* mutations (49). We therefore labeled PMCA2 in the hair bundles of the *Tmc1* mutants (Fig. 8 *A* and *B*). Immunofluorescence density was reduced in all mutants (Fig. 8 *C*–*E*), with the degree of reduction increasing from *Tmc1* p.T416K (33 percent) to *Tmc1* p.M412K (56 percent) to *Tmc1* p.D569N (68 percent) (Fig. 8*F*). The reduction in PMCA2 density for the three mutants was closely correlated with the decrease in the Ca^2+^ permeability of the MET channel in the mutant compared to wild-type *Tmc1^+/+^* (Fig. 8*G*).

**Fig. 9.**
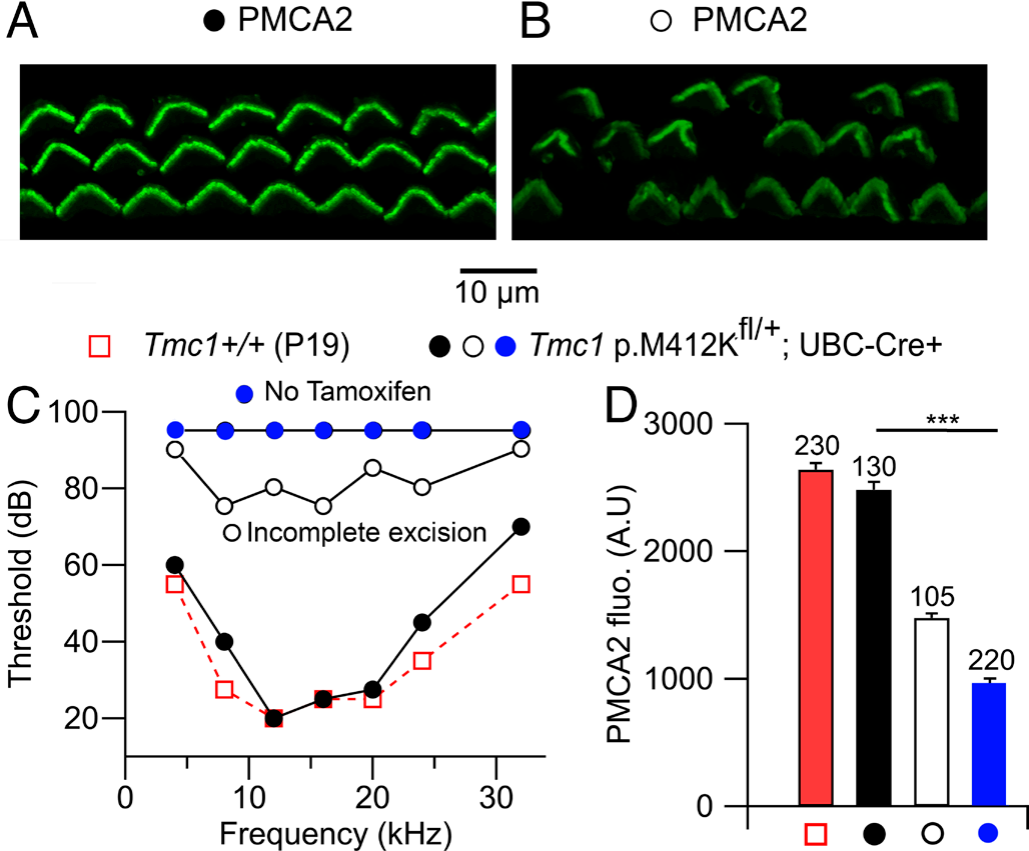
PMCA2 expression in *Tmc1* M412K Cre-lox mutants. (*A*) PMCA2 label in P19 *Tmc1* p.M412K fl/+; UBC-Cre/+ with full ABR recovery. (*B*) PMCA2 label in P16 *Tmc1* p.M412K fl/+; UBC-Cre/+ with incomplete recovery. Note the distortion of hair bundle shape and the missing bundles. (*C*) ABR thresholds for *Tmc1+/+* (control, red squares), *Tmc1* p.M412K fl/+; UBC-Cre/+ with full recovery (fill circles), *Tmc1* p.M412K fl/+; UBC-Cre/+ with no tamoxifen (blue circles), and *Tmc1* p.M412K fl/+; UBC-Cre/+ with incomplete excision (open circles). (*D*) PMCA2 counts for the four experiments, numbers of OHCs measured being indicated above bars. Control and full recovery are not significantly different. All measurements on organs of Corti from middle turns.

The relationship between PMCA2 expression and hearing was examined further by PMCA2 labeling in those mice where the mutant M412K exon had been excised at P1 (Fig. 9). Of the three mice studied, two had good hearing but one showed a poor ABR threshold. Cochleas from one with a good threshold and one with a poor threshold (Fig. 9*C*) were isolated and immunolabeled. The mouse with good recovery exhibited uniform “V” shaped OHC bundles all of which were well labeled (Fig. 9*A*). In contrast, OHCs from the mouse with poor hearing had deformed hair bundles and holes in the organ of Corti, such that 22 percent of the bundles were missing (Fig. 9*B*), presumably because the OHCs had undergone apoptosis. The PMCA2 density of the recovered mouse was identical to the control *Tmc1+/+* mouse of the same age, P19 (Fig. 9*D*), but for the one with poor recovery, the PMCA2 density was reduced by 42 percent. A *Tmc1* p.M412K/+; UBC-Cre-ERT2/+ mouse without tamoxifen injection had an even larger 63 percent reduced PMCA2 density (Fig. 9*D*). These results demonstrate a correlation between stereociliary PMCA2 density, hair cell apoptosis, and deafness.

## Discussion

The aim of our work was to clarify the metabolic pathway which cochlear hair cells traverse during apoptosis, triggered by a point mutation in *Tmc1*. We have previously shown that with such mutations, the MET channels are gated normally at P6 (12). For the three mutations studied here, two (*Tmc1* p.M412K and *Tmc1* p.T416K) display maximal MET currents at P6 indistinguishable from controls, but the third (*Tmc1* p.D569N), had a reduced current amplitude due to a defect in transporting TMC1 to its activation site at the lower end of the tip link (23). However, by the third postnatal week, transduction in OHCs of the cochlear apex is lost and when tested at 4 wk of age the mice of all three mutants are deaf and many OHCs, especially those at the base, have disappeared (7). We focused on OHCs because in those *Tmc1* mutants, OHCs but not IHCs show apoptosis by P28 (7, 23).

We have found that even at P6, there are manifestations of early apoptosis, revealed by three markers: Annexin V labeling for scramblase activity, reduced Calcein-AM labeling due to loss of cytoplasmic demethylating enzymes, and mitochondrial dysfunction, due to loss of mitochondrial membrane potential and assumed mitophagy. We conclude that the initial controlling factor is an increase in stereociliary [Ca^2+^], based on the observation that inhibiting Ca^2+^ extrusion by PMCA2 initiates scramblase activity (Fig. 7). Intracellular Ca^2+^ is set by the balance between the efflux and influx of the ion, and we found that reducing external Ca^2+^ from 1.5 to 0.5 mM during PMCA2 inhibition reduced scramblase activation. Our conclusion is opposite to that previously suggested (17), which was largely based on the reduced MET channel Ca^2+^ permeabilities of *Tmc1* deafness mutants that initiate scramblase activity. We propose the following apoptotic pathways:

MET mutation → increased stereociliary [Ca^2+^] → (i) scramblase activation and (ii) mitochondrial depolarization → compromising mitochondrial integrity. The second (ii) pathway is probably more important as mitochondrial malfunction will diminish ATP production, further increasing intracellular [Ca^2+^] because of reduced PMCA2 extrusion. Additionally, there is evidence (Fig. 2*B*) that the scramblase pathway is less important and may not be required for apoptosis.

The main intrinsic apoptotic pathway proceeds via Bcl2 or Bcl6 signals (21, 22). Two prominent BCL2 proapoptotic factors are BAK and BAX, which under cellular stress are recruited from the cytoplasm. The complex acts to permeabilize the outer mitochondrial membrane, evoking mitochondrial depolarization, release of cytochrome C, and activation of a series of caspases culminating in cell death. Support for the contribution of mitochondrial malfunction in hair cell apoptosis was the effects of *Bak; Bax* double knockouts to partially mitigate loss of auditory sensitivity in *Tmc1* p.D569N mutants (Fig. 4*E*). It was previously found that reduction in BAX alleviated the apoptotic effects of treatment with aminoglycosides in zebrafish lateral line hair cells (50). Besides the apoptotic pathway we describe, there are other processes occurring consequent on hair cell mutations or assault, such as thinning or deformation of the stereociliary tips. These have been reported after block of the MET channels (51, 52) and in later development of *Tmc1* p.D569N, figure 3E in ref. 23. The stereociliary effects of exposure to blockers may be connected with actin polymerization, possibly mediated by the action of Ca^2+^ on gelsolin (53) and in the short term be reversible (51).

The conclusion that the hair cell apoptosis pathway in *Tmc1* mutants was triggered by an increase in cytoplasmic Ca^2+^, although consistent with the mode of operation of the TMEM16F scramblase, presents a paradox since the three mutants studied all generate MET channels having reduced Ca^2+^ permeability. Furthermore, the apparent severity of the mutants, as implied by the time of onset of deafness, is proportional to the degree of reduction in Ca^2+^ permeability, the *Tmc1* p.T416K retaining some hearing function longer than the *Tmc1* p.M412K. A way out of the paradox is the finding that the PMCA2 pump density was significantly diminished in the mutants. The causal factor linking *Tmc1* mutation to reduced pump density is not known for certain. A homeostatic mechanism, reduced PMCA2 density because of decreased Ca^2+^ influx through mutant MET channels, may be one explanation, and is supported by the correlation between the two (Fig. 8*G*). PMCA2 expression in rodent hair bundle develops over the first postnatal week, in parallel with the MET current, the base preceding the apex by about 3 d (30). This would be consistent with Ca^2+^ influx via the MET channels driving expression of the pump. The hair bundle PMCA2 shows rapid turnover in the first few postnatal days (54) and it is possible that during this early period, PMCA2 insertion into the stereociliary membrane is regulated by stereociliary [Ca^2+^]. In this case, the fractional PMCA2 density is expected to correlate with the MET channel Ca^2+^ permeability relative to *Tmc1*+/+ (Fig. 8*G*). The link was supported by the finding that early postnatal excision of the mutant exon promoted recovery of both auditory threshold and PMCA2 density in the stereocilia.

There are precedents for the effects of PMCA2 mutations on hair cell function. In the *deafwaddler^2J^* mutation (55), there is a DNA frameshift producing a truncated protein that is not expressed in the stereocilia (55) and is associated with deafness. However, in two point mutations, *Oblivion* (48) and *Tommy* (49), the mutant protein is still present in the hair bundle, but it pumps more slowly. For example, in the *Obl/Obl* homozygote, there is a 2.5-fold slower Ca^2+^ extrusion after cytoplasmic Ca^2+^ uncaging, and the homozygote shows severe hearing loss. Thus, a reduced pumping rate causes a substantial hearing deficit. A comparable loss of pumping might result from the smaller PMCA2 expression in the *Tmc1* mutants. Additionally, in PMCA2 mutants, there is a base to apex degeneration of the OHCs by P40 (49), implying that the basal OHCs are more sensitive to defects in Ca^2+^ balance, which may account for their greater vulnerability (56). Thus, a diminished ability to extrude Ca^2+^ could have more severe consequences in smaller basal OHCs with higher Ca^2+^ turnover.

## Data Availability

All study data are included in the main text.
